# Circular RNA YAP1 inhibits the proliferation and invasion of gastric cancer cells by regulating the miR-367-5p/p27 ^Kip1^ axis

**DOI:** 10.1186/s12943-018-0902-1

**Published:** 2018-10-18

**Authors:** Hui Liu, Yuan Liu, Zhaolian Bian, Jing Zhang, Rui Zhang, Xiaoyu Chen, Yanxia Huang, Yang Wang, Jinshui Zhu

**Affiliations:** 1 Department of Gastroenterology, Shanghai Jiao Tong University Affiliated Sixth People’s Hospital, No. 600 Yishan Road, Shanghai, 200233 China; 2 Nantong Institute of Liver Disease, Department of Gastroenterology and Hepatology, Nantong Third People’s Hospital, Nantong University, Nantong, 226006 Jiangsu China; 3 Medical School of Nantong University, Nantong, 226006 Jiangsu China

**Keywords:** circYAP1, Gastric cancer, Growth, Invasion, miR-367-5p

## Abstract

**Background:**

Circular RNAs (circRNAs) are a new type of non-coding RNAs and their functions in gastric cancer (GC) remain unclear. Recent studies have revealed that circRNAs play an important role in cancer development and certain types of pathological responses, acting as microRNA (miRNA) sponges to regulate gene expression.

**Methods:**

CircNet was used to screen potential circRNAs and validated circYAP1 expression levels in 17 GC tissues by quantitative real-time PCR (qRT-PCR) and another 80 paired GC tissues by FISH. CircYAP1 overexpression and knockdown experiments were conducted to assess the effects of circYAP1 in vitro and in vivo, and its molecular mechanism was demonstrated by RNA in vivo precipitation assays, western blotting, luciferase assay and rescue experiments.

**Results:**

CircYAP1 expression level was significantly lower in GC tissues than the adjacent normal tissues, and GC patients with circYAP1 low expression had shorter survival times as compared with those with circYAP1 high expression. Functionally, circYAP1 overexpression inhibited cell growth and invasion in vitro and in vivo, but its knockdown reversed these effects. Further analysis showed that circYAP1 sponged miR-367-5p to inhibit p27 ^Kip1^ expression and GC progression.

**Conclusion:**

Our findings demonstrate that circYAP1 functions as a tumor suppressor in GC cells by targeting the miR-367-5p/p27 ^Kip1^ axis and may provide a prognostic indicator of survival in GC patients.

**Electronic supplementary material:**

The online version of this article (10.1186/s12943-018-0902-1) contains supplementary material, which is available to authorized users.

## Background

Gastric cancer (GC) continues to be a major threat to human health and it is the fourth most common cancer and the third-leading cause of cancer-related deaths worldwide according to global cancer statistics [1]. Despite the application of many advances in diagnosis and treatment, the prognosis of GC remains relatively poor, with a 5-year overall survival below 40% in most countries, due to tumor metastasis and recurrence [2]. In the past decades, non-coding RNAs (ncRNAs), including microRNA (miRNA) and long non-coding RNA (lncRNA) have been deregulated in GC patients, and have potential clinical applications [3, 4]. Recent studies have shown that circular RNAs (circRNAs) are aberrantly expressed in GC, lung cancer, hepatocellular carcinoma (HCC) and colorectal cancer (CRC), involved in cancer development [5]. Therefore, it is essential to identify deregulated circRNAs and discover novel molecular mechanisms and therapeutic targets for the treatment of GC.

CircRNAs are a special type of ncRNAs derived from exons, introns or intergenic regions that are covalently linked to form a closed circular structure without 5′ caps and 3′ tails, display cell or tissue-specific expression, and are conserved across species due to their resistance to RNase R [6–8]. Compared with linear RNAs, circRNAs are remarkably stable, and accumulate primarily in the cytoplasm, acting crucial roles in human diseases [9, 10]. Emerging evidence shows that circRNAs act as miRNA sponges to regulate gene expression and interact with RNA binding proteins (RBPs) [8, 11]. However, the functions of the newly identified circRNAs in special fields require further investigation.

CircRNAs participate in a wide range of biological processes, including transcription, mRNA splicing, RNA decay and translation, and their dysregulation leads to abnormal cellular functions and human diseases [12]. It is revealed that certain types of circRNA are deregulated in HCC, CRC, esophageal squamous cancer, oral cancer and bladder cancer, and are associated with cancer progression [13–17]. Those studies indicate that circRNAs may be potential biomarker and therapeutic target for cancer.

In our study, we selected a circRNA, termed circYAP1 (has_circ_0002320) by CircNet (http://syslab5.nchu.edu.tw/CircNet/) and validated that circYAP1 expression level was dramatically decreased in GC tissues. Low expression of circYAP1 was associated with the poor prognosis of patients with GC. More importantly, we found that circYAP1 functioned as a sponge of oncogenic miR-367-5p to upregulate p27 ^Kip1^ and consequently suppressed the tumorigenesis of GC.

## Methods

### Clinical data and tissues

A human tissue microarray of 80 paired GC patients (Cat No. STC1602) was purchased from Shanghai Superbiotek Pharmaceutical Technology Co. Ltd. (Shanghai, China). Seventeen GC and paired adjacent gastric tissue samples were obtained from patients undergoing surgery at Nantong Third People’s Hospital Affiliated to Nantong University. All samples were collected with consent from the patients and were stored at − 80 °C until use. All experiments were approved by the Ethics Committee of Shanghai Jiao Tong University Affiliated Sixth People’s Hospital and Nantong Third People’s Hospital Affiliated to Nantong University.

### Identification of miRNAs sponged by circYAP1

The miRNAs that might be sponged by circYAP1 were identified by using CircNet and Circular RNA Interactome, of which the top 3 miRNAs (miR-367-5p, miR-513c-3p and miR-513a-3p) sponged by circYAP1 were selected through the CircNet, and another 3 miRNAs (miR-1200, miR-330-5p, and miR-513a-3p) were chosen by the Circular RNA Interactome. Therefore, miR-513a-3p could be predicted to sponge circYAP1 by both CircNet and Circular RNA Interactome, and these miRNAs were further confirmed by qPCR in circYAP1 overexpressed cells.

### RNA fluorescence in situ hybridization (FISH)

An oligonucleotide-modified probe sequence for human circYAP1 (3’-CCTCTTCCTCTCCGACGCCGACTTTGTCGTTCTTGACGAAGCCGTCCAGGAGAAGGACTACCTACCCTTGTTCGGTACTGAGTCCTACCTCTTTA-5′) was used for FISH. The probe of circYAP1 was marked with DIG-UTP (Roche,11,209,256,910) for RNA labeling. After dehydration with 70, 95 and 100% ethanol, hybridization was carried out at 37 °C overnight in a dark moist chamber. After hybridization, slides were washed three times in 50% 60 ml formamide/2X SSC for 5 min and was incubated with anti-DIG-HRP (PerkinElmer, NEF832001EA) at 4 °C overnight, after being washed for 3 times for 10 min at room temperature, the slides were incubated with TSA fluorescent signal reaction solution (PerkinElmer, NEL701001KT, TSA Fluorescein system) for 30 min and was sealed with tablets containing DAPI. The images were acquired using a fluorescence microscopy (Leica, SP8 laser confocal microscopy). The analysis software Image-pro plus 6.0 (Media Cybernetics, Inc., Rockville, MD, USA) was applied to acquire the Immunofluorescence Accumulation Optical Density (IOD) for evaluating the expression level of circYAP1 in GC tissues.

### CircYAP1 and miR-367-5p co-location by RNA fluorescence in situ hybridization (FISH)

The probe for circYAP1 was 5′-FAM-ATCAG GAAGA GGACC TGCCG AAGCA GTTCT-FAM-3′, and the probe for miR-367-5p was 5′-CY3-AGAGT TGCAT ATTAG CAACA GT-CY3–3′. After dehydration with 70, 95 and 100% ethanol, hybridization was carried out at 37 °C overnight in a dark moist chamber. After each hybridization, slides were washed three times in 50% 60 ml formamide/2X SSC for 5 min respectively. The images were acquired using a fluorescence microscopy (Leica, SP8 laser confocal microscopy). The analysis software Image-pro plus 6.0 (Media Cybernetics, Inc., Rockville, MD, USA) was applied to acquire the Immunofluorescence Accumulation Optical Density (IOD) for evaluating the expression level of circYAP1 and miR-367-5p in GC tissues.

### Cell culture

The normal human gastric epithelial cell line GES-1 and a GC cell line (HGC-27) were purchased from the Cell Laboratory of Central South University. MKN-45 and AGS cell lines were obtained from the Digestive Disease Laboratory of Shanghai. GES-1 and GC cell lines were cultured in Dulbecco’s modified Eagle’s medium (DMEM) (Gibco, Rockford, MD, USA) or 1640 medium (Gibco) supplemented with 10% fetal bovine serum (FBS), 100 U/ml penicillin, and 100 μg/ml streptomycin. All cells were cultured in a humidified atmosphere containing 5% CO_2_ at 37 °C.

### Quantitative real-time PCR (qRT-PCR)

Total RNA was extracted using RNAiso Plus (TaKaRa, Dalian, China), reverse transcription was performed using PrimeScrip™ RT Master Mix (TaKaRa), and cDNA amplification was performed using SYBR Green Premix Ex Taq™ II (Takara) according to the manufacturer’s instructions. miRNA was extracted using an miRNeasy Mini Kit (Qiagen, Duesseldorf, Germany), reverse transcription was performed using an miScript II RT Kit (Qiagen), and cDNA amplification was performed using an miScript SYBR Green PCR Kit (Qiagen) according to the manufacturer’s instructions. The primers are listed in Additional file 1: Table S1.

### CircRNA in vivo precipitation (circRIP)

A biotin-labeled circYAP1 probe was designed and synthesized by GenePharma. The sequence was as follows: 5′-GAA CTG CTT CGG CAG GTC CTC TTC CTG A-3′-biotin; the circRIP assay was performed according to the reported literature with minor alterations [13, 18]. CircYAP1-overexpressing AGS cells were seeded in a 10-cm dish at a density that allowed them to grow for 48 h without reaching complete confluency. Then, the cells were transfected with the specific biotin-tagged probe or control probe at a final concentration of 200 nmol/L. The cells were harvested after transfection for 24 h. Then, the cells were cross-linked with 1% formaldehyde for 10 min, lysed and sonicated. After centrifugation, 50 μL of the supernatant was retained as input, and the remaining cell lysis solution was incubated with a circYAP1-specific probe-streptavidin Dynabeads (M-280, Invitrogen, CA, USA) mixture overnight at room temperature. The next day, the M-280 Dynabead-probe-circRNA mixture was washed and incubated with 200 μL of lysis buffer and proteinase K to reverse the formaldehyde crosslinking. Finally, the mixture was extracted to obtain the total RNA using the miRNeasy Mini Kit according to the manufacturer’s instructions (Qiagen).

### Lentiviral vector, siRNA, mimics and inhibitors

The lentivirus-mediated circYAP1 overexpression vector and no-load lentivirus vector were supplied by Hanbio (Shanghai, China). GC cells (MKN-45 and AGS) were infected with the lentivirus according to the manufacturer’s instructions, and stable cells were selected with puromycin. miR-367-5p mimics and inhibitors were synthesized by GenePharma (GenePharma, Suzhou, China). The siRNA sequence that targeted circYAP1 for knockdown was as follows: CGG CAG GUC CUC UUC CUG ATT; this sequence was designed and synthesized by GenePharma. The mimics, inhibitor and siRNA were transfected with Lipofectamine 2000 (Invitrogen) according to the manufacturer’s instructions.

### Cell proliferation assay

Cell proliferation was detected using a CCK-8 assay kit (Dojindo Corp, Japan). A total of 3000 cells were plated in each well of a 96-well plate. Then, on the indicated day, 10 μL of CCK-8 reagent was added directly to the culture medium. Then, the cells were incubated for 2.5 h at 37 °C, and the optional density was measured at 450 nm. These experiments were repeated three times.

### 5-Ethynyl-20-deoxyuridine (EdU) incorporation assay

EdU assays were performed using a Cell-Light EdU DNA Cell Proliferation Kit (RiboBio, Guangzhou, China) according to the manufacturer’s instructions. A total of 1 × 10^4^ cells were seeded in each well of a 96-well plate. After incubation with 50 μM EdU for 2 h, the cells were fixed in 4% paraformaldehyde and stained with Apollo Dye Solution. Hoechst-33,342 was used to stain the nucleic acids. Images were acquired using an Olympus IX73 microscope (Olympus, Tokyo, Japan), and the percentage of EdU-positive cells was calculated using ImageJ (National Institutes of Health, Bethesda, MD, USA). These experiments were repeated three times.

### Colony formation assay

After MKN-45 and AGS cells were transfected with the indicated lentivirus, mimics, inhibitor or siRNA for 48 h, a total of 1 × 10^3^ cells were seeded in a 6-well plate and incubated at 37 °C for 7 days, and the medium was changed every two days. On day 7, all the cells were fixed in 4% paraformaldehyde and dyed with a crystal violet solution. Cell colonies were then counted and analyzed. These experiments were repeated three times.

### Transwell invasion assay

The cell invasion assay was carried out using 24-well Transwell (Corning Costar, 8.0 μm pore size) insert chamber plates. The upper surfaces of Transwell filters were coated with Matrigel (BD, New Jersey, USA). Transfected cells (3 × 10^5^) in 200 μl of serum-free 1640 medium were added to the upper compartment of the chamber. A total of 500 μL of 1640 medium supplemented with 10% FBS was added into the lower chamber. The invaded cells were harvested after incubation for 48 h. The non-invaded cells on the upper side of the chamber were scraped off with a cotton swab. The cells were fixed in 4% paraformaldehyde and stained with a crystal violet solution. The cells were then counted and analyzed. These experiments were repeated three times.

### Western blotting analysis

MKN-45, AGS and HGC-27 cells were harvested and extracted using RIPA lysis buffer (Beyotime, Shanghai, China), and equal amounts of cells extracts were separated on 12% SDS-PAGE gels. The separated protein bands were transferred onto nitrocellulose (NC) membranes. The primary antibodies anti-p27^Kip1^ (Cell Signaling Technology, MA, USA) and anti-β-actin (Proteintech, IL, USA) were diluted 1:1000 according to the instructions and incubated overnight at 4 °C. HRP rabbit IgG secondary antibodies were added at a dilution of 1:2000 and incubated for 1 h at room temperature. The membranes were washed three times with TBST, and the immunoreactive bands were visualized using SuperSignal West Dura Extended Duration Substrate (Thermo Fisher, IL, USA) according to the kit’s instructions. These experiments were repeated three times.

### Luciferase reporter assay

293T cells were seeded into 96-well plates. The fragments including the 3′-UTR wild-type (P27-WT) regions of p27^Kip1^ were cloned into SacI/SalI-digested pmirGLO vector (Promega, USA), which included both Renilla and Firefly luciferase reporter genes. The mutant p27^Kip1^ 3′-UTR (P27-MUT) was generated by mutating the conserved binding sites for miR-367-5p using Gene Mutation Kit (Takara, JAPAN). Then 60 ng P27-WT or P27-MUT plasmid were co-transfected with miR-367-5p mimics into 293T cells. The miR-NC mimics was used as negative control. After 48 h, the cells were harvested, and the Firefly and Renilla luciferase activities were measured with a dual-luciferase reporter assay system (Promega, USA). The relative luciferase activity was normalized to Renilla luciferase activity.

### Flow cytometric analysis

To detect cell apoptosis, GC cells were digested, washed with cold PBS and resuspended in binding buffer according to the instructions of the apoptosis kit. APC-AnnexinV and 7-AAD were added to the fixed cells for 15 min in the dark at room temperature. Then, Annexin V binding buffer was added to the mixture before the fluorescence was measured with a BD FACSCalibur flow cytometer (BD). Cell apoptosis was analyzed using Cell Quest software (Becton Dickinson, USA). Three separate experiments were performed.

To detect cell cycle, cells were seeded in a six-well plate at 5 × 10^5^ cells per well. After transfection, the cells were maintained for 48 h before harvest. Harvested cells were washed with PBS and fixed with 70% ethanol. The fixed cells were stained with PI in the presence of RNase A for 15 min at room temperature in the dark. The samples were then analyzed with a BD FACSCalibur flow cytometer (BD) after acquiring 10000 events for each sample. FlowJo V10 software was used for the cell cycle analyses. Three separate experiments were performed.

### Tumor model in mice

NOD/SCID mice were purchased from the Shanghai Laboratory Animal Central (SLAC, Shanghai, China). Approval for the experiments was obtained from the Institutional Animal Care and Use Committee of Shanghai Jiao Tong University Affiliated to Shanghai Sixth People’s Hospital. MKN-45 cells (1 × 10^7^) transfected with the circYAP1 overexpression vector or no-load lentivirus vector were resuspended in PBS and injected subcutaneously into the right front armpits of 6-week-old mice. After 4 weeks, the mice were sacrificed, and the xenografted tumors were collected to calculate the volume and for further experiments.

### Statistical analysis

Statistical analyses were carried out using SPSS 20.0(IBM, SPSS, Chicago, IL, USA) and GraphPad Prism (GraphPad, La Jolla, USA). Student’s t-test or Chi-square test was used to assess the statistical significance for comparisons of two groups. OS was defined as the interval between the dates of surgery and death, and OS curves were analyzed with the Kaplan-Meier method and log-rank test. Univariate analysis and multivariate models were performed using a Cox proportional hazards regression model. Receiver operating characteristic (ROC) curves were obtained using cutoff Finder online software (http://molpath.charite.de/cutoff/load.jsp). *P* < 0.05 was considered statistically significant.

## Results

### Low expression of circYAP1 indicates poor prognosis in GC patients

**Fig. 1:**
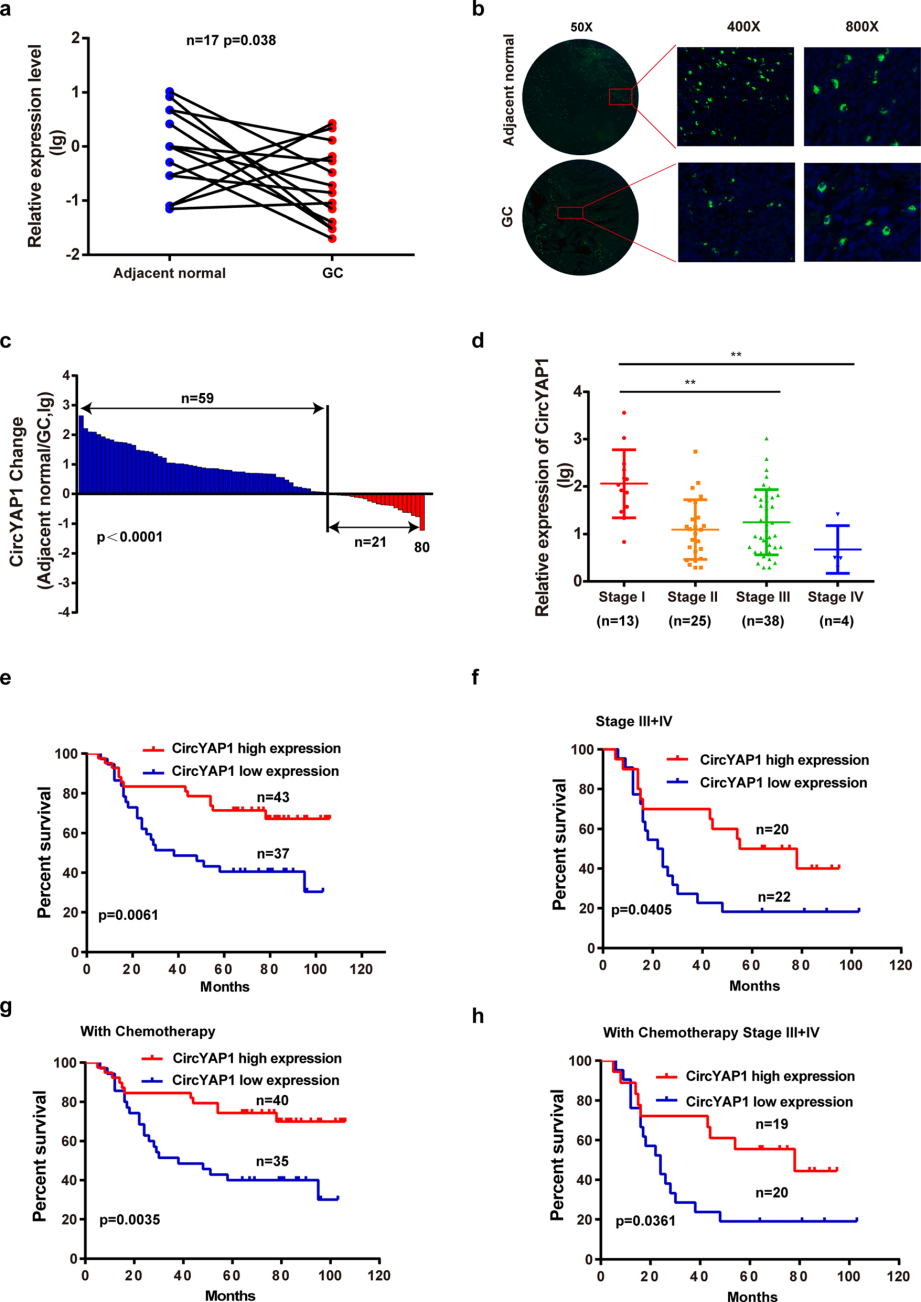
CircYAP1 acts as an independent prognostic factor for OS in GC patients. **a**, CircYAP1 expression was significantly lower in GC tissues than in normal adjacent tissues from 17 patients with GC according to qPCR. **b**, Cellular localization of circYAP1 in GC tissue cells and adjacent normal tissue cells. The nuclei was stained blue with DAPI, and cytoplasmic circYAP1 was stained green. **c**, CircYAP1 expression levels were lower in GC tissues than in normal tissues. **d**, CircYAP1 expression levels were higher in early-stage GC patients than in advanced stage GC patients. **e-f**, Kaplan-Meier analysis of the association of circYAP1 expression levels with the OS of GC patients and late-stage GC patients (stage III + IV). **g**-**h**, Kaplan-Meier analysis of the association of circYAP1 expression with the therapeutic outcomes of GC patients and late-stage GC patients (stage III + IV). *P < 0.05; **P < 0.01

We measured the expression of circYAP1 in 17 patients with GC by qPCR. Compared with that in adjacent normal tissues, circYAP1 expression was significantly decreased from 1 to 0.09 in 76.5% (13 of 17) of GC tissues (Fig. 1a, *P* = 0.0383). We further confirmed this result by FISH assay using tissue microarray in 80 GC samples from patients with survival data (Additional file 1: Table S2). As shown in Fig. 1b, circYAP1 was dramatically lower in GC tissues than in adjacent normal tissues. In addition, we found that circYAP1 expression was lower in GC patients with a tumor size (TS) ≥ 5 cm than those with a TS < 5 cm (Additional file 2: Figure S1a). Compared with that in GC tissue, the circYAP1 expression level in adjacent normal tissue was higher in 59 GC patients and lower in 21 GC patients (Fig. 1c, *P* < 0.0001). To illustrate the relationship between circYAP1 and GC, we divided the GC patients into four groups according to the TNM staging system. CircYAP1 expression was dynamically altered with the GC stage, and it was higher in patients with early-stage GC than those with advanced stage GC (Fig. 1d).

Next, we analyzed whether circYAP1 can act as an independent prognostic factor for GC patients. The GC patients were divided into two groups, circYAP1 high expression and circYAP1 low expression, according to the cutoff value, which was defined by Cutoff Finder (Additional file 2: Figure S1b). High circYAP1 expression was negatively associated with tumor size but was not correlated with other pathological parameters (Additional file 1: Table S3).

Survival analyses were performed using log-rank tests. GC patients with high circYAP1 expression had longer overall survival (OS) times than those with low circYAP1 expression (Fig. 1e, *P* = 0.0061). In addition, advanced stage (stage III + IV) patients with high circYAP1 expression had longer OS times than those with low circYAP1 expression (Fig. 1f, *P* = 0.0405). Furthermore, we analyzed the association between circYAP1 expression levels and therapeutic outcomes in 75 GC patients treated with adjuvant chemotherapy (oxaliplatin and 5-Fu). We found that the GC patients with high circYAP1 expression had more favorable therapeutic outcomes than those with low circYAP1 expression (Fig. 1g, *P* = 0.0035). The same result was observed for the advanced stage patients (Fig. 1h, *P* = 0.0361). To determine whether circYAP1 is an independent prognostic factor of OS in GC patients, univariate and multivariate Cox proportional hazards analyses were performed. CircYAP1 level, tumor size and lymphatic metastasis were independent prognostic factors for the OS of GC patients (Additional file 1: Table S4).

### CircYAP1 was confirmed to sponge MiR-367-5p in GC cells

**Fig. 2:**
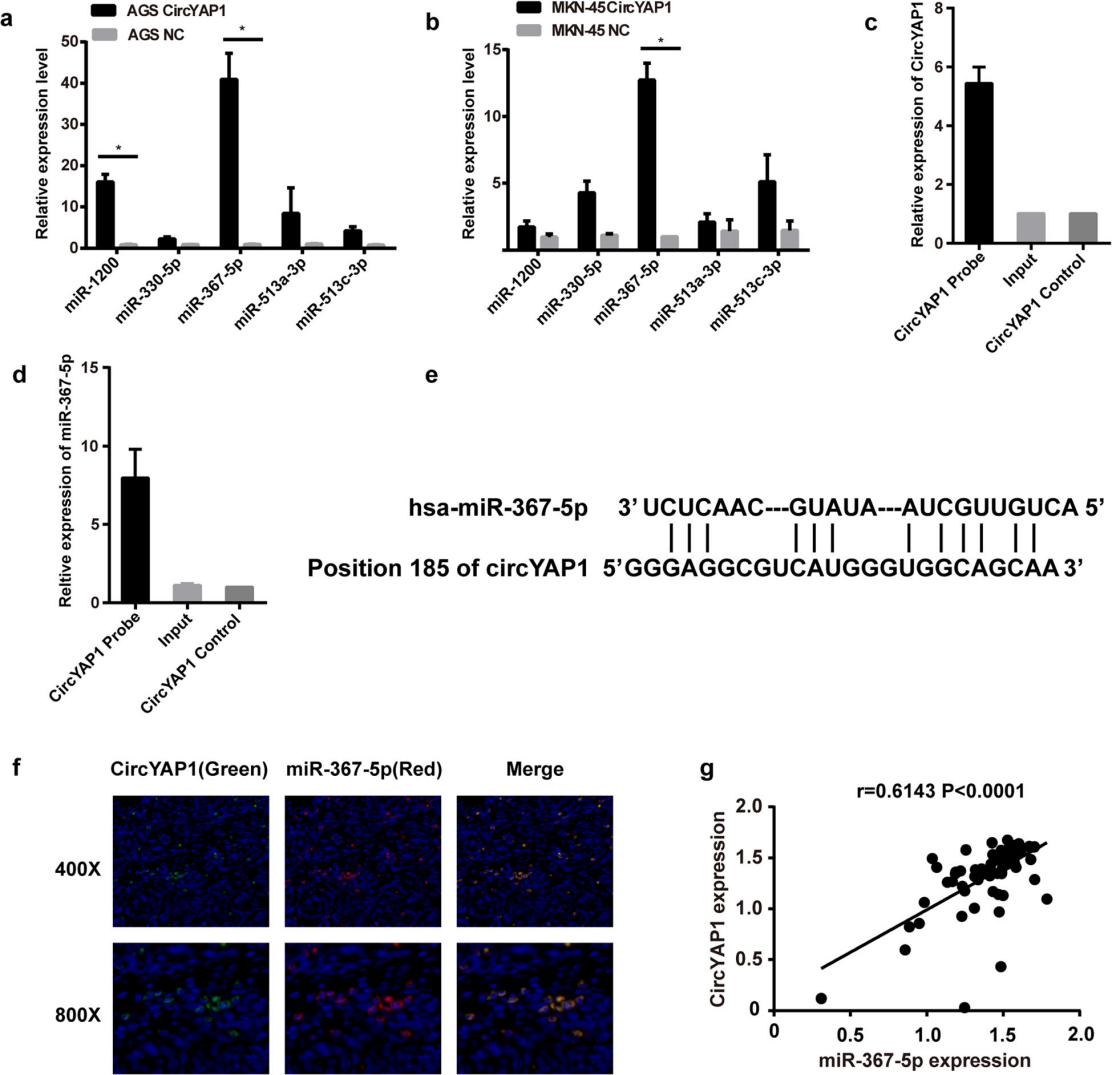
Identification of the potential miRNAs sponged by circYAP1. **a-b**, miRNA expression in circYAP1-overexpressing AGS and MKN-45 cells. **c-d**, CircYAP1 in GC cell lysates was pulled down and enriched with a circYAP1-specific probe and then detected by qPCR. **e**, Schematic representation of the potential binding sites of miR-367-5p with circYAP1. **f**, circYAP1 was co-localized with miR-367-5p in the cytoplasm in GC tissues. **g**, Pearson correlation analysis of the correlation of miR-367-5p with circYAP1 expression in human GC tissues

As previously reported, circRNAs primarily function as miRNA sponges to regulate gene expression. Therefore, we examined the potential miRNAs associated with circYAP1. CircInteractome and CircNet were used to predict the potential target miRNAs that could bind with the circYAP1 sequence, and five miRNAs were selected as the best potential targets of circYAP1. To investigate whether these five miRNAs can interact with circYAP1 in GC cells, we first overexpressed circYAP1 in the AGS and MKN-45 cell lines and performed qPCR to measure the following miRNAs: miR-1200, miR-330-5p, miR-367-5p, miR-513a-3p and miR-513c-3p. The results showed that miR-1200 and miR-367-5p were significantly upregulated by circYAP1 in the AGS cells (*P* = 0.0161; *P* = 0.0238), and the expression levels of the other miRNAs were not changed (Fig. 2a). In addition, only miR-367-5p expression was increased by circYAP1 in the MKN-45 AGS cells (Fig. 2b, *P* = 0.0113). Therefore, miR-367-5p might be bound to circYAP1 in GC cells. To confirm this hypothesis, we used a circYAP1-specific probe to perform RNA in vivo precipitation (RIP) which has been reported in several studies [13, 18]. We purified circYAP1-associated RNA using a circYAP1 specific-probe and performed qPCR to measure the expression of circYAP1 and miR-367-5p. qPCR showed the enrichment of circYAP1 and miR-367-5p compared to the controls (Fig. 2c and d, *P* = 0.0050; *P* = 0.0224), indicating that miR-367-5p was sponged by circYAP1 in GC cells. The binding sites of miR-367-5p to circYAP1 were shown in Fig. 2e. By using FISH analysis in GC tissues, we found that circYAP1 and miR-367-5p were co-localized in the cytoplasm (Fig. 2f). The expression of circYAP1 had the positive correlation with miR-367-5p in GC tissues (Fig. 2g, *r* = 0.641, *P* < 0.0001). These results suggested that circYAP1 might bind with much more miR-367-5p, resulting in the decrease of free miR-367-5p.

### CircYAP1 inhibits the proliferation and invasion of GC cells

To investigate the biological functions of circYAP1 in GC cells, we performed qPCR to measure the expression level of circYAP1 in GC cells. CircYAP1 levels were lower in GC cells than GES-1 cells. AGS cells showed the lowest expression level, MKN-45 cells showed a moderate expression level, and HGC-27 cells showed the highest expression level (Additional file 3: Figure S2a). Therefore, AGS and MKN-45 cells were chosen for circYAP1 overexpression, and HGC-27 cells were selected for circYAP1 silencing. We constructed circYAP1-overexpressing lentiviruses with circular frames and circYAP1 sequences and found that circYAP1 was significantly overexpressed in AGS and MKN-45 cells (Additional file 3: Figure S2b and c). Moreover, miR-367-5p mimics were successfully transfected into the AGS and MKN-45 GC cells (Additional file 3: Figure S2d and e). In addition, the circYAP1 expression level transfected with miR-367-5p mimics was similar to those transfected with mimics NC (Additional file 3: Figure S2f).

**Fig. 3:**
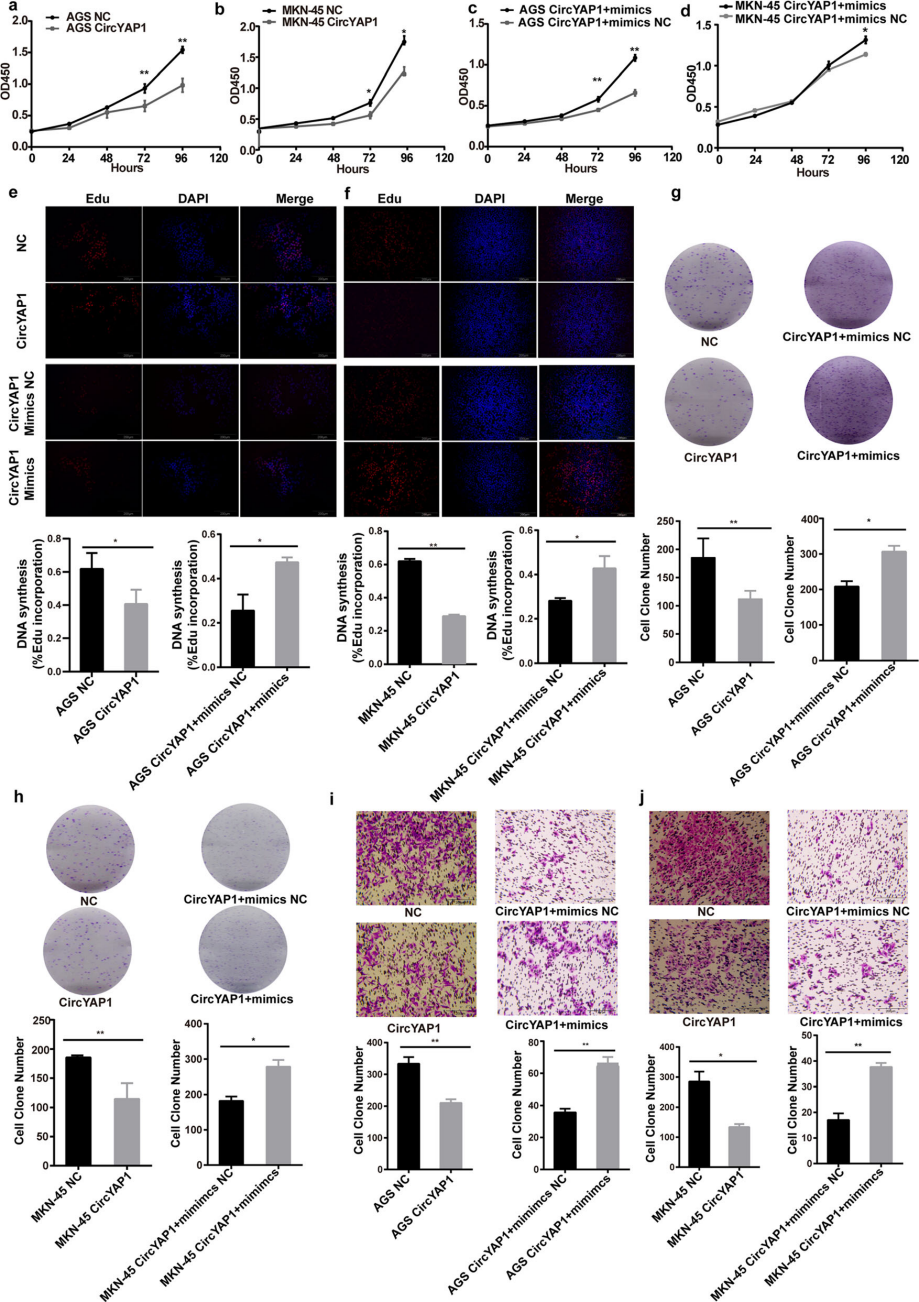
CircYAP1 inhibits proliferation, colony formation and invasion. **a-d**, Cell proliferation activity and **e-f** DNA synthesis were assessed in AGS and MKN-45 cells transfected with the circYAP1 overexpression vector or circYAP1 overexpression vector + miR-367-5p mimics. **g-h**, Clone formation assays were conducted in AGS and MKN-45 cells transfected with the circYAP1 overexpression vector or circYAP1 overexpression vector + miR-367-5p mimics. **i-j**, Cell invasion potential was determined in AGS and MKN-45 cells transfected with the circYAP1 overexpression vector or circYAP1 overexpression vector + miR-367-5p mimics according to Transwell assays. *P < 0.05; **P < 0.01

The effects of circYAP1 overexpression on cell proliferation, invasion and cycle distribution were determined by 5-ethynyl-2′-deoxyuridine (EdU), clone formation, Transwell assays and flow cytometry analysis. Cell proliferation assay showed that circYAP1 overexpression significantly suppressed AGS and MKN-45 cell growth (Fig. 3a and b). Cell cycle distribution assays also revealed that circYAP1 overexpression induced considerable arrest at the G1 phase in AGS and MKN-45 cells (Additional file 4: Figure S3a and b, *P* = 0.0038; *P* = 0.0156). However, the effects of cell proliferation suppression and cell cycle arrest induced by circYAP1 were reversed when the cells were co-transfected with miR-367-5p mimics (Fig. 3c-d and Additional file 4: Figure S3a and b). EdU incorporation assays showed that GC cells proliferation was impaired upon circYAP1 overexpression (Fig. 3e and f, *P* = 0.0172; *P* < 0.0001), and this impairment could be rescued by co-transfection with the miR-367-5p mimics (Fig. 3e and f, *P* = 0.0264; *P* = 0.0353). Furthermore, clone formation assay indicated that the compromised colony-forming ability of these two GC cell lines upon circYAP1 overexpression could return to normal levels when the cells were co-transfected with miR-367-5p mimics (Fig. 3g and h). In addition, the invasion ability of these two types of GC cells was impaired upon circYAP1 overexpression, and this impairment could be rescued by the miR-367-5p mimics (Fig. 3i and j). However, cell apoptosis was not affected by circYAP1 in GC cells (Additional file 2: Figure S1c-f).

### CircYAP1 knockdown promotes GC cell proliferation and invasion

**Fig. 4:**
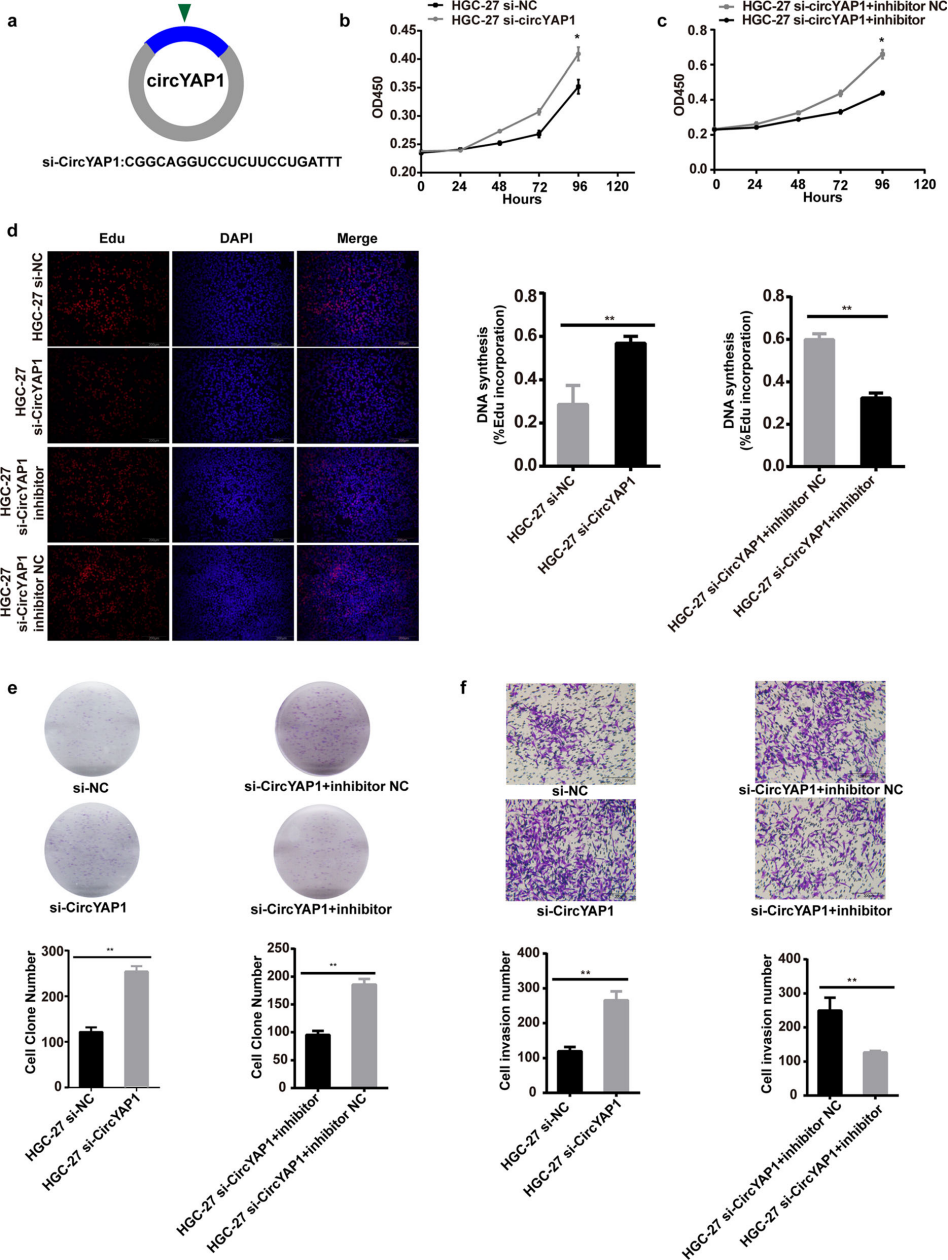
Knockdown circYAP1 promotes proliferation, colony formation and migration. **a**, Schematic representation of the target sequences of the siRNAs specific to the back-splicing junction of circYAP1. **b-c**, Cell proliferation activity and **d**, DNA synthesis in HGC-27 cells transfected with si-circYAP1 or si-circYAP1 + miR-367-5p inhibitor. **e**, Clone formation assays of HGC-27 cells transfected with si-circYAP1 or si-circYAP1 + miR-367-5p inhibitor. **f**, Cell invasion potential of HGC-27 cells transfected with si-circYAP1 or si-circYAP1 + miR-367-5p inhibitor according to Transwell assays. *P < 0.05; **P < 0.01

To evaluate the negative biological functions of circYAP1, we used small interference RNA (siRNA) that targeted the back-splice sequence of circYAP1 in HGC-27 cells (Fig. 4a). The siRNA efficiency was confirmed by qPCR (Additional file 3: Figure S2 g). Cell proliferation assays revealed that cell proliferation was enhanced upon circYAP1 silencing (Fig. 4b) and these effects could be ablated by the miR-367-5p inhibitor (Fig. 4c). Cell cycle assays indicated that the proportion of G1 and G2/M cells in the phase decreased upon circYAP1 silencing (Additional file 4: Figure S3c, *P* = 0.0010; *P* = 0.0003). However, the proportion of cells in the S phase increased when the cells were co-transfected with miR-367-5p inhibitor (Additional file 4: Figure S3c, *P* = 0.0002; *P* = 0.0003). EdU incorporation assays showed that cell proliferation was enhanced upon circYAP1 knockdown (Fig. 4d, *P* = 0.0064) and these effects could be neutralized by the miR-367-5p inhibitor (Fig. 4d, *P* = 0.0002). Clone formation assays revealed that circYAP1 knockdown promoted the colony-forming ability of HGC-27 cells (Fig. 4e, *P* = 0.0056), but these effects were weakened when the cells were co-transfected with the miR-367-5p inhibitor (Fig. 4e, *P* = 0.0004). Furthermore, cell invasion assays showed that the invasion ability of HGC-27 was enhanced by circYAP1 knockdown (*P* = 0.0009), but this effect was reversed by co-transfected with the miR-367-5p inhibitor (Fig. 4f, *P* = 0.0050).

### P27^Kip1^ was validated as a target gene of miR-367-5p

**Fig. 5:**
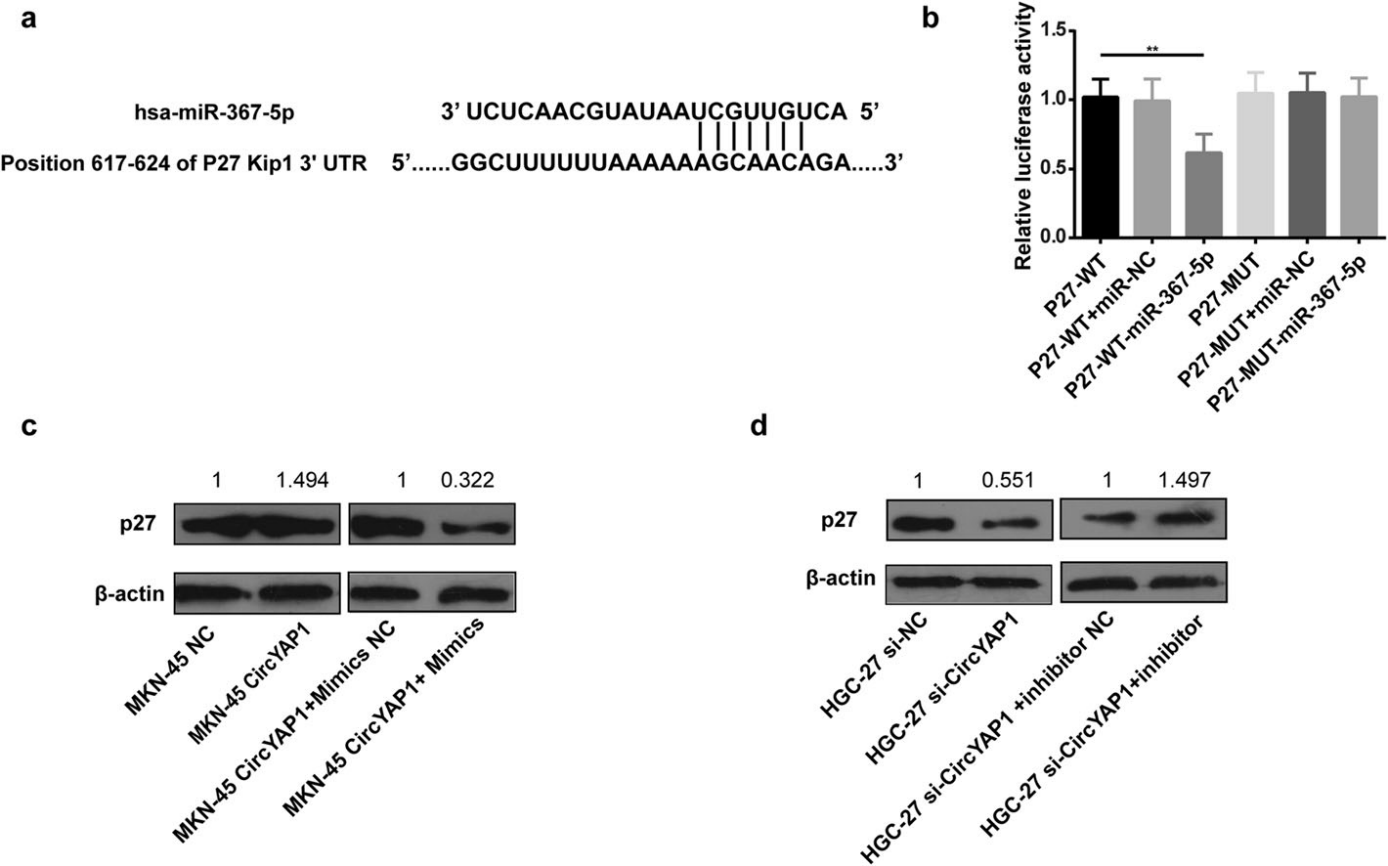
P27 ^Kip1^ was the target of miR-367-5p in GC cells. **a**, Schematic representation of the potential binding sites of miR-367-5p with p27 ^Kip1^. **b**, double luciferase activity in 293T cells. **c**, p27 ^Kip1^ expression levels in MKN-45 cells after transfection with the circYAP1 overexpression vector or circYAP1 overexpression vector + miR-367-5p mimics. **d**, p27 ^Kip1^ expression levels in HGC-27 cells transfected with si-circYAP1 or si-circYAP1 + miR-367-5p inhibitor. *P < 0.05; **P < 0.01

Next, we used Targetscan (http://www.targetscan.org/vert_71/) to predict the target genes of miR-367-5p; and the gene gene-p27 ^Kip1^ had the best potential (Fig. 5a). 293T cells co-transfected with plasmid containing 3′-UTR-WT regions of p27 ^Kip1^ and miR-367-5p mimics had significantly less relative luciferase activity than the controls, while mutation of the potential miR-367-5p binding sites in the p27 ^Kip1^ 3′-UTR abolished the effect (Fig. 5b, *p* = 0.0029). It was indicated that p27 ^Kip1^ might be a putative target of miR-367-5p.

### CircYAP1 sponged miR-367-5p to upregulate p27 ^Kip1^

To determine whether circYAP1 sponges miR-367-5p to regulate p27 ^Kip1^ expression, we measured p27 ^Kip1^ expression level by Western Blotting [19–22]. CircYAP1 overexpression considerably upregulated p27 ^Kip1^ expression in GC cells, and this effect could be reversed by miR-367-5p mimics (Fig. 5c). In addition, silencing circYAP1 decreased the expression of p27 ^Kip1^ and this effect could be also rescued by the miR-367-5p inhibitor (Fig. 5d).

### CircYAP1 inhibits tumor growth in vivo

**Fig. 6:**
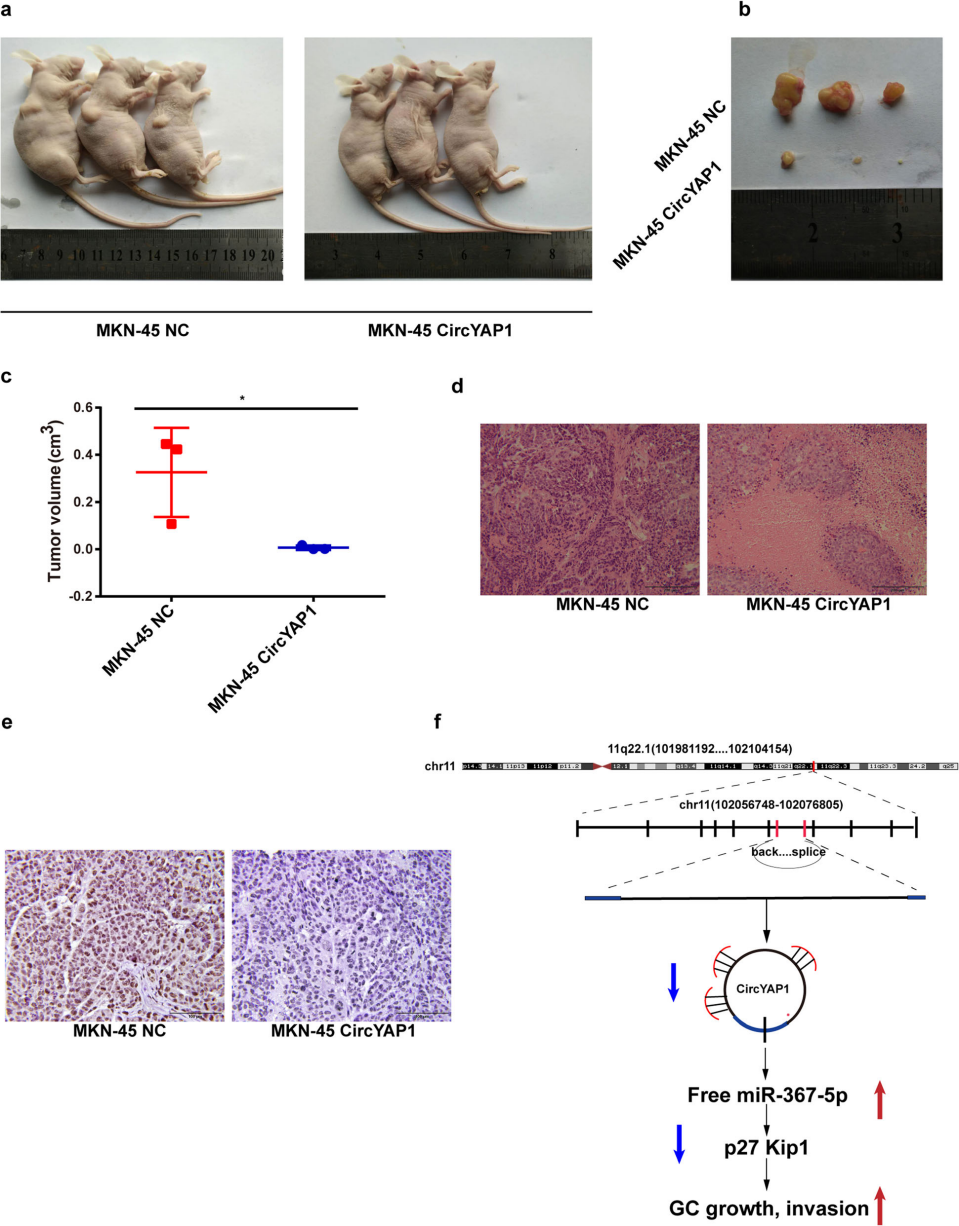
CircYAP1 inhibited tumor growth in vivo. **a**, Nude mice were subcutaneously injected with circYAP1-overexpressing MKN-45 cells and NC cells. **b**, Schematic representation of the MKN-45 xenograft tumor size after 30 days of tumor growth in the circYAP1 overexpression and NC groups. **c**, Statistical comparison of the differences in tumor volumes between the circYAP1 overexpression and NC groups. **d**, HE analysis of the tumor formation in circYAP1 overexpression and NC groups. **e**, IHC analysis of Ki-67 expression levels in xenograft tumor tissues from the circYAP1 overexpression and NC groups (× 400). **f**, Loss of circYAP1 sponges and increases the free miR-367-5p levels, and thereby decreased p27 ^Kip1^ expression, leading to the GC tumorigenesis

To further explore whether circYAP1 influences tumor growth in vivo, we constructed circYAP1 overexpression or negative control (NC) stably transfected MKN-45 cells were subcutaneously injected into the flank of nude mice. After 30 days, the tumor volumes were significantly lower in circYAP1-overexpressing MKN-45 cells than in NC-transfected cells (Fig. 6a, b). Statistical analyses showed that the tumor volume was much smaller in the circYAP1-overexpressing group than in the NC-transfected group (Fig. 6c, *P* = 0.0432). HE staining showed that the tumor formation ability of circYAP1-overexpressing group was lowered as compared with the NC-transfected group (Fig. 6d). IHC analyses showed that the Ki-67 expression was decreased by circYAP1 overexpression (Fig. 6e).

## Discussion

CircRNAs are a new group of ncRNAs with largely unclear biological functions. The roles of circRNAs in carcinogenesis have recently attracted extensive attention. It has been reported that circRNAs are aberrantly expressed in tumors and function as tumor suppressors or oncogenes in the progression of cancer pathogenesis [5, 23, 24]. For example, circCCDC66, circMTO1, circMYLK and circHIAT1 promote tumor growth and metastasis [13, 14, 16, 25], while circLARP4 and circZKSCAN1 suppress tumor progression by multiple complicated signaling pathways [26, 27]. Here, we combined the reported literature with a CirNet analysis to find circYAP1, which is derived from the YAP1 gene locus [28]; Many studies have revealed that YAP1 enhances cell proliferation, migration and invasion in GC [29, 30]. However, the expression and functional role of circYAP1 in GC remain unknown. We investigated 17 GC tissues and paired adjacent normal tissues and found that circYAP1 expression was significantly higher in adjacent normal tissues than in matched GC tissues. It was suggested that circYAP1 might act as an anti-oncogene rather than an oncogene in GC. This result was further verified in another 80 cases of GC tissues.

Decreased circYAP1 expression in GC was significantly correlated with poor prognosis in GC patients. GC patients with high circYAP1 expression had longer survival times than those with low circYAP1 expression. CircRNA expression was dynamically changed with the development of the disease. CircYAP1 expression levels were higher in the early-stage GC patients than in the late-stage GC patients. In addition, GC patients with high circYAP1 expression levels were more sensitive to chemotherapy than those with low circYAP1 expression levels. It was illustrated that circYAP1 could act as a biomarker for predicting the survival or an index for chemotherapy responses in GC patients.

CircRNAs in different types of cancer or different stages vary in regulating the gene expression [31]. CircRNAs are well known to serve as miRNA sponges to form the circRNA-miRNA-mRNA axis [8, 32]. For example, our previous study revealed that circLARP4 sponges miR-424-5P to activate LATS1-YAP signaling in GC [27]. CircCCDC66 sponges multiple miRNAs to promote CRC growth and metastasis [14]. CircMOT1 sponges miR-9 to suppress HCC progression [13], circMYLK sponges miR-29a to activate the VEGFA/VEGFR2 pathway to promote bladder cancer progression [16], and ciRS-7 is involved in a variety of cancers by its interaction with miR-7 [33].

In this study, CircNet and CircInteractome were used to predict circRNAs [34], and the circRNAs were selected with the relatively unbiased approach of computational algorithms. As reported. ciRS-7 is resistant to the conventional miRNA destabilization of mRNA and therefore not prone to miR-7-dependent regulation. miR-7 and ciRS-7 display co-expression in cells. The high expression of ciRS-7 coincides with miR-7 expression in mouse brain sections and in primary cells isolated from mouse brain, which strongly suggests that miR-7 is interacting endogenously with ciRS-7. CDR1as has been proposed to function as a sponge for miR-7 by reducing the number of freely available miR-7 molecules [8]. Other studies also revealed that the loss of circRNAs causes miRNA degeneration [35]. Thus, we measured the expression levels of the miRNAs with the potential to bind with circYAP1 and identified that miR-367-5p was significantly upregulated by circYAP1 in GC cells. Then, we performed circRIP using a circYAP1-specific probe and found that miR-367-5p was the circYAP1-binding miRNA. We proved that circYAP1 sponged miR-367-5p in GC cells. Further functional experiments showed that circYAP1 inhibited GC cell growth, invasion and cycle progression in vitro and in vivo, but knockdown of circYAP1 had the opposite effects, suggesting that circYAP1 might act as a tumor suppressor in GC.

MiRNA, a key component of the ncRNA family, plays multifaceted roles in controlling cellular functions by repressing target genes [36]. miR-367 has been reported as an oncogene in uveal melanoma cells [37], osteosarcoma [38], medulloblastoma [39] and non-small cell lung cancer [40, 41]. In our study, we also found that miR-367-5p acted as an oncogene in GC cells. p27 ^Kip1^ is a cyclin-dependent kinase (CDK) inhibitor that regulates cell proliferation, cell motility and apoptosis. Low expression of p27^Kip1^ is correlated with high-grade tumors and poor prognosis in several types of human cancer [42]. More importantly, studies also described an association between p27^Kip1^ loss and poor prognosis in gastric cancer [20, 43]. CircYAP1 upregulated the expression level of p27 ^Kip1^, and this effect was abolished by co-transfection with miR-367-5p. CircYAP1 knockdown suppressed p27 ^Kip1^ expression, and the effects could be reversed when the cells were co-transfected with a miR-367-5p inhibitor. The luciferase activity assay also exhibited the binding of miR-367-5p with p27^Kip1^. It was demonstrated that p27^Kip1^ is the target of miR-367-5p and circYAP1 sponges miR-367-5p to upregulates p27 ^Kip1^ expression in GC cells.

## Conclusions

In summary, our findings reveal that circYAP1 expression is significantly decreased in GC and low circYAP1 expression is associated with a poor prognosis in GC patients. Functionally, circYAP1 inhibits GC cell growth and invasion by sponging miR-367-5p to upregulate p27 ^Kip1^. We speculate the loss of circYAP1 sponges less miR-367-5p and increases the free miR-367-5p in GC cells, and thereby decreases p27 ^Kip1^expression, contributing to the GC tumorigenesis (Fig. 6f). CircYAP1 may have considerable potential as a prognostic predictor and therapeutic target for GC.

## Additional files

Additional file 1: **Tables S1.** Sequences of primers in the study. **Table S2.** Clinic-pathological data of GC patients from Tissue Microarray. **Table S3.** Correlation of circYAP1 expression with clinic-pathologic characteristics of GC patients. **Table S4.** Summary of univariate and multivariate Cox regression analysis of overall survival duration (DOCX 20 kb)

Additional file 2: **Figure S1. a,** The expression levels of circYAP1 in GC patients with TS ≥5 cm or <5 cm. **b,** Receiver operating characteristic (ROC) curve analysis of the cutoff value, sensitivity, specificity and AUC of circYAP1 in GC patients. **c-f,** Flow cytometry was used to detect the proportion of apoptotic cells in circYAP1-overexpression-transfected MKN-45 cells (PDF 241 kb)

Additional file 3: **Figure S2**. **a,** CircYAP1 expression in GES-1 and GC cell lines. **b-c,** AGS and MKN-45 GC cells transfected with the circYAP1 overexpression lentivirus. **d-e,** miR-367-5p mimics were transfected into AGS and MKN-45 GC cells. **f,** qPCR analysis of the transfection efficiency of si-circYAP1 vectors after transfection for 48 h in HGC-27 cells. *P < 0.05; **P < 0.01 (PDF 619 kb)

Additional file 4: **Figure S3.** Cell cycle analysis. **a,** Cell cycle assays of AGS transfected with circYAP1 or circYAP1 + miR-367-5p mimics. **b** Cell cycle assays of MKN-45 transfected with circYAP1 or circYAP1 + miR-367-5p mimics. **c** Cell cycle assays of HGC-27 cells transfected with si-circYAP1 or si-circYAP1 + miR-367-5p inhibitor. *P < 0.05; **P < 0.01 (PDF 1324 kb)

## Abbreviations

circRIP: CircRNA in vivo precipitation
circRNA: Circular RNA
EdU: 5-Ethynyl-20-deoxyuridine
GC: gastric cancer
miRNA: MicroRNA
qRT-PCR: Quantitative real-time polymerase chain reaction
YAP1: Yes-associated protein 1
