# Correction to: Circular RNA YAP1 inhibits the proliferation and invasion of gastric cancer cells by regulating the miR-367-5p/p27 Kip1 axis

**DOI:** 10.1186/s12943-019-1045-8

**Published:** 2019-07-09

**Authors:** Hui Liu, Yuan Liu, Zhaolian Bian, Jing Zhang, Rui Zhang, Xiaoyu Chen, Yanxia Huang, Yang Wang, Jinshui Zhu

**Affiliations:** 1 Department of Gastroenterology, Shanghai Jiao Tong University Affiliated Sixth People’s Hospital, No. 600 Yishan Road, Shanghai, 200233 China; 2 Nantong Institute of Liver Disease, Department of Gastroenterology and Hepatology, Nantong Third People’s Hospital, Nantong University, Nantong, 226006 Jiangsu China; 3 Medical School of Nantong University, Nantong, 226006 Jiangsu China

**Correction to: Mol Cancer**

**https://doi.org/10.1186/s12943-018-0902-1**

After the publication of this work [1], a spelling error was found: the ID of the circRNA, termed circYAP1, in the original publication was misspelled as “has_circ_0002320”. The corrected spelling is “hsa_circ_0002320”. We sincerely apologize for the spelling error; however, it does not affect any of the interpretations or conclusions of the article.
