# Supplementary material for: Circular RNA YAP1 inhibits the proliferation and invasion of gastric cancer cells by regulating the miR-367-5p/p27 Kip1 axis
Source: Mol Cancer. 2018 Oct 18;17:151. doi: 10.1186/s12943-018-0902-1 (PMC6193296; doi:10.1186/s12943-018-0902-1)
Supplement: Supplementary file 1 — Tables S1. Sequences of primers in the study. Table S2. Clinic-pathological data of GC patients from Tissue Microarray. Table S3. Correlation of circYAP1 expression with clinic-pathologic characteristics of GC patients. Table S4. Summary of univariate and multivariate Cox regression analysis of overall survival duration (DOCX 20 kb) [file 12943_2018_902_MOESM1_ESM.docx]

| Genes | Forward primer | Reverse primer |
| --- | --- | --- |
| circYAP1 | 5’- ACAGATGCGACTGCAGCAAC-3’ | 5’- TGGGTCTAGCCAAGAGGTGG-3’ |
| miR-1200 |  | 5’-CTCCTGAGCCATTCTGAGCCTC-3’ |
| miR-330-5p |  | 5’- CTCTGGGCCTGTGTCTTAGGC-3’ |
| miR-367-5p |  | 5’cgcgACTGTTGCTAATATGCAACTCT-3’ |
| miR-513a-3p |  | 5’- ccgcgTAAATTTCACCTTTCTGAGAAGG-3’ |
| miR-513c-3p |  | 5’cgcgcgTAAATTTCACCTTTCTGAGAAGA-3’ |

Tables S1 Primers

Table S2 Clinicopathological data of GC patients from Tissue Microarray

| Parameters | Cases n (%) |
| --- | --- |
| Total | 80 (100.00%) |
| ***Age (y)*** |  |
| ≥60 | 46 (57.50%) |
| <60 | 34 (42.50%) |
| ***Gender*** |  |
| Male | 56 (70.00%) |
| Female | 24 (30.00%) |
| ***Tumor size (cm)*** |  |
| ≥5 | 38 (47.50%) |
| <5 | 42 (52.50%) |
| ***Pathological classification*** |  |
| Adenocarcinoma (AC) | 60 (75.00%) |
| Signet ring cell carcinoma (SRCC) | 15 (18.75%) |
| AC+SRCC | 5 (6.25%) |
| ***Pathological stage*** |  |
| Ⅰ-Ⅱ | 22 (27.5%) |
| Ⅱ-III | 20 (25.0%) |
| III | 38 (47.5%) |
| ***T classification*** |  |
| T1-T2 | 24 (30.00%) |
| T3-T4 | 56 (70.00%) |
| ***Lymph node status*** |  |
| Negative | 31 (38.75%) |
| Positive | 49 (61.25%) |
| ***Distant metastasis*** |  |
| Negative | 75 (93.75%) |
| Positive | 5 (6.25%) |
| ***With chemotherapy*** |  |
| Negative | 5 (6.25%) |
| Positive | 75 (93.75%) |

Table S3 Correlation of circYAP1 expression with clinicopathologic characteristics of GC patients

| Variables | Cases  (n) | circYAP1 | | *P* value |
| --- | --- | --- | --- | --- |
|  |  | High | Low |  |
| Total | 80 | 43 | 37 |  |
| ***Age (y)*** |  |  |  |  |
| ≥60 | 46 | 25 | 21 |  |
| <60 | 34 | 18 | 16 | 0.901 |
| ***Gender*** |  |  |  |  |
| Male | 56 | 30 | 26 |  |
| Female | 24 | 13 | 11 | 0.961 |
| ***Tumor size (cm)*** |  |  |  |  |
| ≥5 | 38 | 15 | 23 |  |
| <5 | 42 | 28 | 14 | 0.015^*^ |
| ***Pathological classification*** |  |  |  |  |
| Adenocarcinoma (AC) | 60 | 33 | 27 |  |
| Signet ring cell carcinoma (SRCC) | 15 | 8 | 7 |  |
| AC+SRCC | 5 | 2 | 3 | 0.625 |
| ***Pathological stage*** |  |  |  |  |
| Ⅰ-Ⅱ | 22 | 10 | 12 |  |
| II-III | 20 | 13 | 7 |  |
| III | 38 | 20 | 18 | 0.439 |
| ***T classification*** |  |  |  |  |
| T1-T2 | 24 | 16 | 8 |  |
| T3-T4 | 56 | 27 | 28 | 0.149 |
| ***Lymph node status*** |  |  |  |  |
| Negative | 31 | 19 | 12 |  |
| Positive | 49 | 24 | 25 | 0.282 |
| ***Distant metastasis*** |  |  |  |  |
| Negative | 75 | 42 | 33 |  |
| Positive | 5 | 1 | 4 | 0.118 |
| ***With chemotherapy*** |  |  |  |  |
| Negative | 5 | 3 | 2 |  |
| Positive | 75 | 40 | 35 | 0.772 |

Table S4 Summary of univariate and multivariate Cox regression analysis of overall survival duration

| Parameter | Univariate analysis | | |  | Multivariate analysis | | |
| --- | --- | --- | --- | --- | --- | --- | --- |
|  | *P* | HR | 95%CI |  | *P* | HR | 95%CI |
| Age (≥60 vs. <60 years) | 0.670 | 1.153 | 0.598-2.224 |  | NA |  |  |
| Gender (Male vs. Female) | 0.993 | 0.997 | 0.492-2.019 |  | NA |  |  |
| Tumor size (≥5 vs. <5 cm) | 0.001 | 2.984 | 1.495-5.960 |  | 0.052 | 0.483 | 0.231-1.007 |
| Pathological classification (AC vs. SRCC vs. AC+SRCC) | 0.026 | 1.675 | 1.052-2.666 |  | 0.213 | 0.508 | 0.137-1.879 |
| Pathological stage (III vs II+ III vs. Ⅰ+Ⅱ) | 0.551 | 1.123 | 0.767-1.643 |  | NA |  |  |
| T classification (T3+T4 vs. T1+T2) | 0.143 | 1.871 | 0.831-3.899 |  | NA |  |  |
| N classification (N2+N3 vs. N0+N1) | 0.000 | 6.606 | 2.544-17.149 |  | 0.000 | 0.173 | 0.065-0.460 |
| Distant metastasis (Positive vs. Negative) | 0.001 | 4.460 | 1.672-11.895 |  | 0.290 | 0.566 | 0.198-1.623 |
| With chemotherapy (Positive vs. Negative) | 0.468 | 0.647 | 0.198-2.116 |  | NA |  |  |
| circYAP1 expression (High vs. low) | 0.011 | 0.432 | 0.221-0.842 |  | 0.045 | 2.043 | 1.017-4.101 |

NA: not analyze
