# Supplementary material for: Circular RNA YAP1 inhibits the proliferation and invasion of gastric cancer cells by regulating the miR-367-5p/p27 Kip1 axis
Source: Mol Cancer. 2018 Oct 18;17:151. doi: 10.1186/s12943-018-0902-1 (PMC6193296; doi:10.1186/s12943-018-0902-1)

Additional file 2: Figure S1. a The expression levels of circYAP1 in GC patients with TS ≥5 cm or <5 cm. b Receiver operating characteristic (ROC) curve analysis of the cutoff value, sensitivity, specificity and AUC of circYAP1 in GC patients. c-f Flow cytometry was used to detect the proportion of apoptotic cells in circYAP1-overexpression-transfected MKN-45 cells.

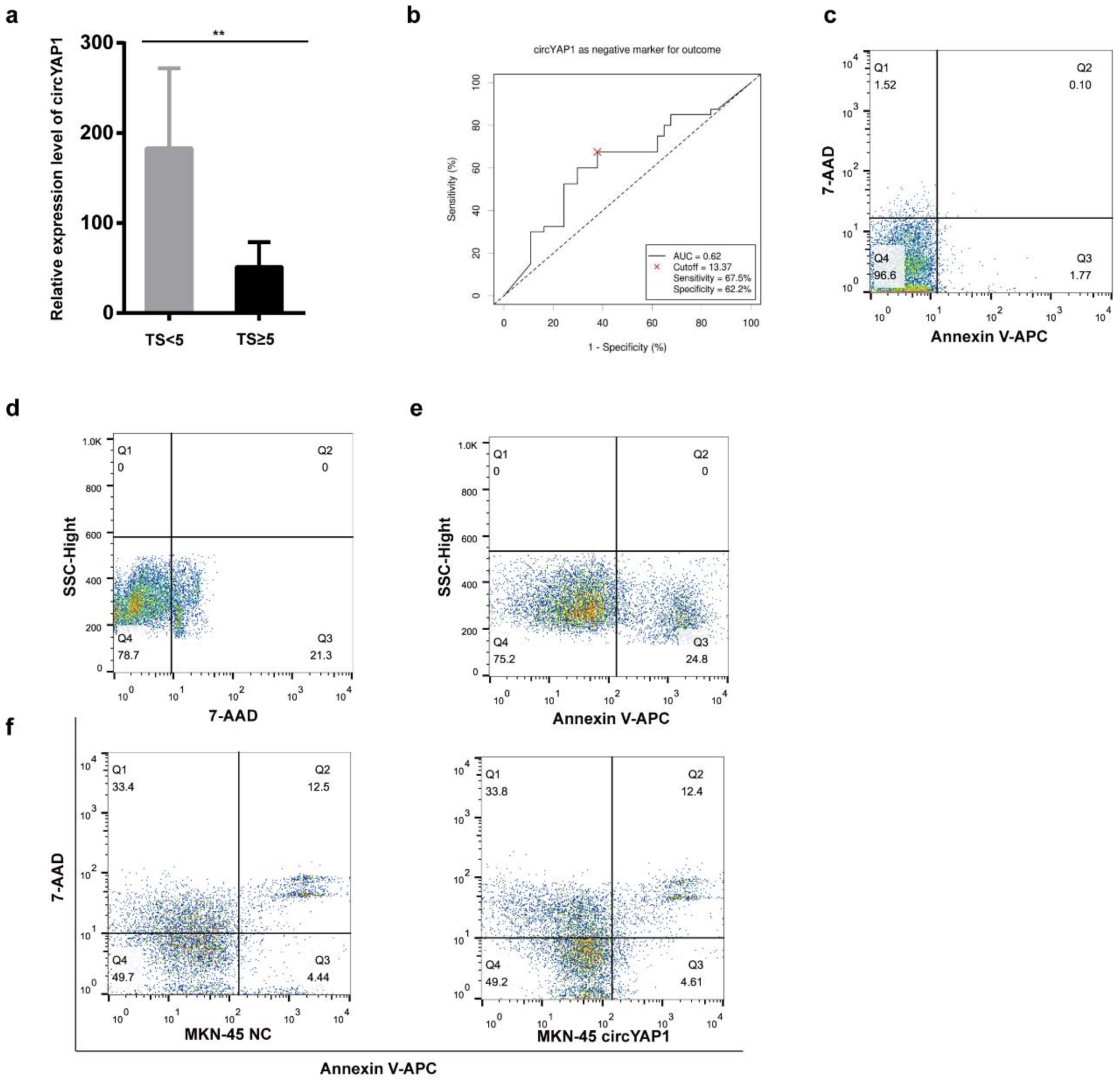

Supplement: Supplementary file 2 — Figure S1. a, The expression levels of circYAP1 in GC patients with TS ≥5 cm or <5 cm. b, Receiver operating characteristic (ROC) curve analysis of the cutoff value, sensitivity, specificity and AUC of circYAP1 in GC patients. c-f, Flow cytometry was used to detect the proportion of apoptotic cells in circYAP1-overexpression-transfected MKN-45 cells (PDF 241 kb) [file 12943_2018_902_MOESM2_ESM.pdf]
