# Supplementary figures and images for: Circular RNA YAP1 inhibits the proliferation and invasion of gastric cancer cells by regulating the miR-367-5p/p27 Kip1 axis
Source: Mol Cancer. 2018 Oct 18;17:151. doi: 10.1186/s12943-018-0902-1 (PMC6193296; doi:10.1186/s12943-018-0902-1)

**a**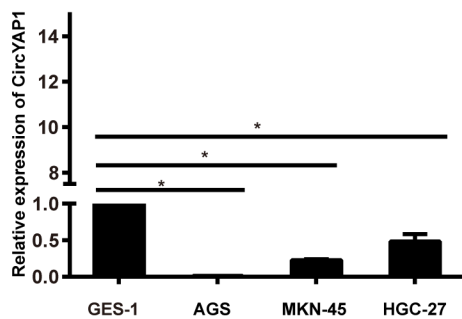**b**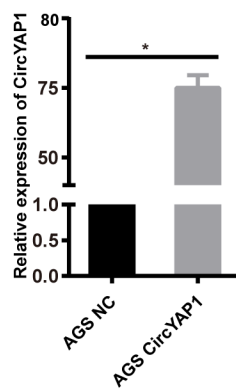**c**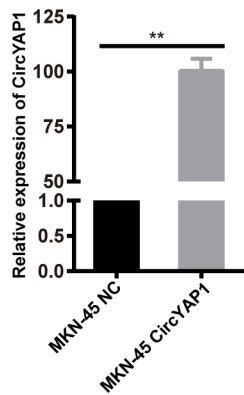**d**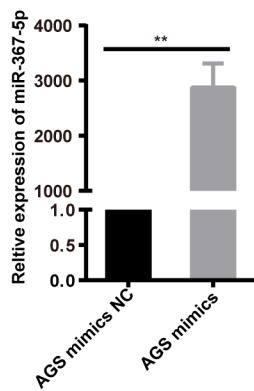**e**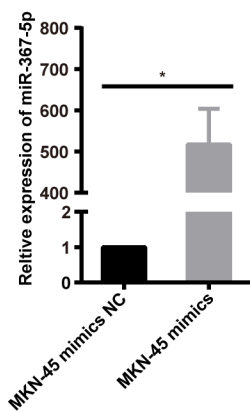**f**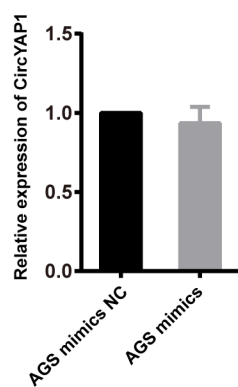**g**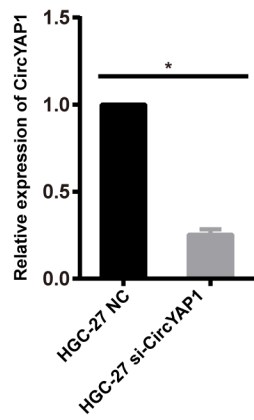

Supplement: Supplementary file 3 — Figure S2. a, CircYAP1 expression in GES-1 and GC cell lines. b-c, AGS and MKN-45 GC cells transfected with the circYAP1 overexpression lentivirus. d-e, miR-367-5p mimics were transfected into AGS and MKN-45 GC cells. f, qPCR analysis of the transfection efficiency of si-circYAP1 vectors after transfection for 48 h in HGC-27 cells. *P < 0.05; **P < 0.01 (PDF 619 kb) [file 12943_2018_902_MOESM3_ESM.pdf]

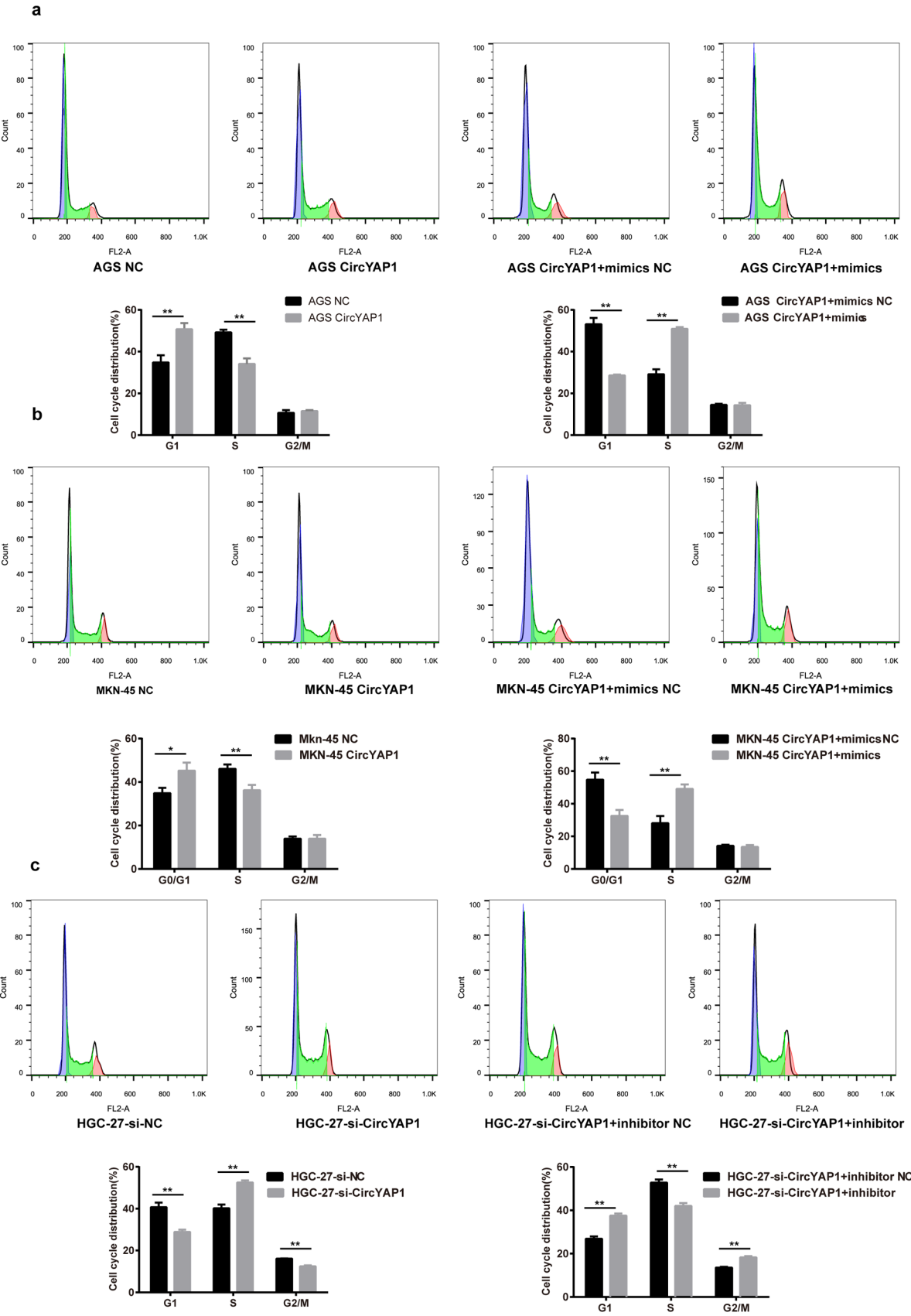

Supplement: Supplementary file 4 — Figure S3. Cell cycle analysis. a, Cell cycle assays of AGS transfected with circYAP1 or circYAP1 + miR-367-5p mimics. b Cell cycle assays of MKN-45 transfected with circYAP1 or circYAP1 + miR-367-5p mimics. c Cell cycle assays of HGC-27 cells transfected with si-circYAP1 or si-circYAP1 + miR-367-5p inhibitor. *P < 0.05; **P < 0.01 (PDF 1324 kb) [file 12943_2018_902_MOESM4_ESM.pdf]
